# Migrant Farmworkers’ Exposure to Pesticides in Sonora, Mexico

**DOI:** 10.3390/ijerph15122651

**Published:** 2018-11-26

**Authors:** Nicolás López-Gálvez, Rietta Wagoner, Paloma Beamer, Jill de Zapien, Cecilia Rosales

**Affiliations:** Mel and Enid Zuckerman College of Public Health, University of Arizona, 1295 N. Martin Ave. PO 245210, Tucson, AZ 85724, USA; rwagoner@email.arizona.edu (R.W.); pbeamer@email.arizona.edu (P.B.); dezapien@email.arizona.edu (J.d.Z.); crosales@email.arizona.edu (C.R.)

**Keywords:** farmworkers, pesticide biomarkers, urine metabolites, occupational health, organophosphates, pyrethroids

## Abstract

Expanding agribusiness in Sonora, a state in Northern Mexico, has increased the demand for temporary migrant agricultural workers. Sonora is one of the top states in Mexico for pesticide utilization. We conducted an exploratory study to evaluate exposure to organophosphate (OP) and pyrethroid pesticides among migrant farmworkers. A sample of 20 migrant farmworkers was recruited from a large commercial grape farm during the harvest season. We administered a questionnaire on work activities, exposure characteristics, and socio-demographics. We collected urine samples to quantify pesticide metabolite concentrations. Most participants were originally from the state of Chiapas, Mexico, none had completed high school, and about half spoke an indigenous language as well as Spanish. The majority of participants had detectable concentrations of pyrethroid and organophosphate biomarkers. Geometric mean creatinine-adjusted concentrations for 3-phenoxybenzoic acid (1.83 µg/g), trans-3-(2,2-dichlorovinyl)-2,2-dimethylcyclopropane carboxylic acid (0.88 µg/g), 4-fluoro-3-phenoxybenzoic acid (0.94 µg/g), 3,5,6-trichloro-2-pyridinol (3.56 µg/g), and para-nitrophenol (0.63 µg/g) were significantly higher than in the general United States’ population and Mexican Americans. Our results also suggest that migrant farmworkers in this region are exposed to pesticides at higher levels than other farmworkers’ studies. Farmworkers’ age, language, training on personal protective equipment, time at the farm, and season, were significant exposure determinants.

## 1. Introduction

According to the Food and Agriculture Organization of the United Nations (FAO), from the year 2004 to 2014, the use of pesticides in Mexico increased from 1.79 kg/ha to 3.85 kg/ha, an increment of 115% [1]. Approximately 50,000 tons of pesticides are used annually in Mexico [2,3]. Use of persistent organochlorine (OCs) pesticides in Mexico and worldwide has gradually been replaced by more degradable pesticides such as organophosphates (OPs) and pyrethroids [4,5]. In Mexico, OPs are among the most commonly used pesticides in urban and rural communities [6,7]. Although OP pesticides are less persistent than OC pesticides, OPs are also toxic and in some cases, even more toxic than OCs [8]. Numerous epidemiological studies have reported associations between OPs and several adverse health outcomes, including Parkinson’s disease and other neurobehavioral effects, birth defects, childhood brain tumors, leukemia, and liver and respiratory issues [4,9,10,11,12,13]. As such, several OPs have been banned or severely restricted worldwide, but they are still being utilized in many urban and rural communities in Mexico [6].

Pyrethroids are another group of synthetic insecticides that are increasingly being used as substitutes in place of OPs. Pyrethroids are widely applied in agriculture, forestry, horticulture, and medical practices, thus, becoming one of the most frequently used class of pesticides [14,15,16]. Research into the health effects of pyrethroids is somewhat limited, yet, some studies have found that pyrethroids may have potential neuro- or developmental toxicity [17,18,19,20,21,22]. Other studies suggest that pyrethroids have estrogenic properties; thus, the United States Environmental Protection Agency (EPA) has listed pyrethroids as possible endocrine-disrupting compounds [23,24,25].

The common use of OP and pyrethroid insecticides in Mexico represents a potential risk for farmworkers’ health. In occupational settings, exposures to pyrethroids and OPs occur mainly from inhalation and dermal exposure. Some of the main barriers to pesticide protection in Mexico include perceptions of low risk from pesticide exposure, lack of management and oversight from all levels, cost, and discomfort of personal protective equipment. Moreover, language and cultural differences are common structural barriers responsible for increasing pesticide exposure of migrant farmworkers in Mexico and the United States (U.S.) [26]. Even though Canada and the U.S. are trying to harmonize their pesticide regulatory system by creating global data dossiers, Mexico is not part of the agreement; as a result, pesticide poisoning is 13 times higher in Mexican workers than U.S. workers [27]. In comparison to other Latin American countries, Mexico has been placed among the countries with the highest rates of diseases related to pesticide exposure among farmworkers [28]. In previous studies, it has been documented that at least 20 percent of seasonal farmworkers in Northern Mexico have experienced acute pesticide poisoning at least once during a season [29].

The large global demand for Mexico’s agricultural products requires an annual movement of approximately over 420,000 workers within Mexico [30], a number that continues to expand along with the growing market. Furthermore, the growing agribusiness in Sonora, a state in Northern Mexico that borders the American states of Arizona and New Mexico, has increased the demand for temporary agricultural workers who migrate from the Southern states of Mexico. Migrant workers from some of the poorest regions of Chiapas, Oaxaca, Puebla, Guerrero, and Veracruz are recruited and contracted in their hometowns [31]. While working at these large-scale commercial farms in Sonora, migrant agricultural workers conduct strenuous tasks while exposed to a wide-ranging array of occupational risks and hazards such as pesticides. In Sonora, as in most regions of Mexico, OPs and pyrethroids are commonly used in agriculture [2].

Although Sonora is among the Mexican states with the highest rates of pesticide usage, there is limited literature on the pesticide exposure in farmworkers in this region [32]. Only two studies have assessed OP exposure in agricultural communities within the state of Sonora [32,33]. With respect to pyrethroids, there are no studies to date that have evaluated the exposure to pyrethroids in farmworkers or any agricultural community in the state of Sonora. Because of the high potential exposures and current lack of data, evaluating exposure to commonly-used pesticides such as OPs and pyrethroids in migrant farmworkers in this region is important.

This exploratory study aimed to characterize the pesticide urine metabolite concentrations in migrant grape workers for the most common agricultural OP and pyrethroid products applied to farms in Sonora, Mexico. This study also seeks to determine potential determinants of pesticide urine metabolite concentrations such as workers’ socio-demographic and occupational characteristics. To the best of our knowledge, this is the first study in this Northern Mexican region to characterize urinary pesticide biomarkers for migrant grape workers exposed to organophosphates and pyrethroids and associated socio-demographic and occupational characteristics.

## 2. Methods

### 2.1. Recruitment

In 2016, we conducted an exploratory cross-sectional study among migrant farmworkers. We recruited a convenience sample of 20 participants from a large commercial grape farm near the central region of Sonora, Mexico that employs approximately 2000 workers, who migrated from other regions in Mexico to live in dormitories within the farm premises during the harvest season. The 20 participants were recruited at the end of the workday on the farm. Only farmworkers who did not apply pesticides were considered for this study. We obtained permission to conduct this study from the farm owner prior to recruitment. A bilingual trained investigator obtained written consent in Spanish from participants. Participants received verbal and written information on the sampling procedure and all sampling equipment in Spanish. Before the initiation of the study, the University of Arizona Human Subjects Protection Program approved all study materials (IRB approval number: 1510159557 on 2 May 2016).

### 2.2. Questionnaire

We orally administered a questionnaire in Spanish to each participant at the conclusion of the workday. The questionnaire was pretested with native Spanish speakers from Sonora, Mexico to check for relevancy and appropriateness. Interviewers were also native Spanish speakers. The questionnaire included sections on socio-demographics (e.g., age, education, home language, state of origin, marital status); potential for occupational pesticide exposure (e.g., experience in agriculture, time working on the farm, work activities directly involving pesticides, knowledge of pesticides applied at the farm and/or in the dormitories); and pesticide protective activities (e.g., training on pesticide safety, personal protective equipment, laundering of work clothes).

### 2.3. Urine Sample Collection

The morning after a regular workday in the field, about 40 mL of urine was collected from each participant in a 50 mL plastic container. Participants were instructed to wash their hands before handling urine containers and to not touch the inside of the containers in order to prevent sample contamination. The samples were kept on ice and transported to the University of Arizona Medical Research Building, where they were stored for less than 48 h at −20 °C until they were shipped to the analytical laboratory.

### 2.4. Urine Sample Laboratory Analysis

Urine samples were analyzed at the National Center for Environmental Health (NCEH) at the Centers for Disease Control and Prevention (CDC) in Atlanta, GA, USA using a modification of the isotope dilution method developed by Reference [34]. In brief, the target pesticide metabolites were extracted and concentrated from urine samples by a semi-automated solid phase extraction technique, separated from each other by reversed-phase high performance liquid chromatography with a gradient elution program, ionized using heated electrospray ionization, and detected by tandem mass spectrometry. Isotopically labeled internal standards were used for the precise and accurate quantitation of the pesticide biomarkers. As presented in Table 1, the urine samples were analyzed for two specific metabolites of OP insecticides: 3,5,6-trichloro-2-pyridinol (TCPY) and para-nitrophenol (PNP); as well as three metabolites of synthetic pyrethroids: 4-fluoro-3-phenoxybenzoic acid (4F3PBA), 3-phenoxybenzoic acid (3PBA), and trans-3-(2,2-dichlorovinyl)-2,2-dimethylcyclopropane carboxylic acid (t-DCCA). The limits of detection (LOD) were 0.1 μg/L (TCPY, PNP, 4FPBA, 3PBA) and 0.6 μg/L (t-DCCA). To normalize metabolite concentrations for varying hydration levels, results were adjusted for creatinine according to the World Health Organization (WHO) guidelines [35]. The urine creatinine measurement for each sample was performed using a Creatinine Assay Kit (R&D Systems, Minneapolis, MN, USA), and analyzed with an EL × 808™ Absorbance Microplate Reader (BiotTek Instruments, Winooski, VT, USA). The involvement of the CDC laboratory was determined not to constitute engagement in human subject research.

### 2.5. Data Analysis

The questionnaire data were transcribed using the Research Electronic Data Capture (REDCap) hosted at the University of Arizona Center for Biomedical Informatics and Biostatistics. REDCap is a secure data collection tool that supports data capture [36]. We performed descriptive statistics of socio-demographic and occupational pesticide exposure characteristics obtained from the questionnaire. We examined the urine metabolite concentrations of organophosphates and pyrethroids using descriptive statistics. Pesticide metabolite concentrations below the LOD were assigned values equivalent to the LOD/$\sqrt{2}$ [37]. We tested associations of urinary metabolites with socio-demographic and occupational pesticide exposure characteristics by using Spearman’s correlation for the continuous variables and the Mann–Whitney *U* test. Because a national assessment of pesticide metabolites in urine conducted for the general population in Mexico is non-existent, we compared the results of this exploratory study to the general U.S. population and Mexican Americans from the 2009–2010 National Health and Nutrition Examination Survey (NHANES). We used the Mann–Whitney U test to determine if our pesticide concentration differed significantly from NHANES dataset (*n* = 2747). All analyses were completed using Stata 12 (StataCorp LP, College Station, TX, USA). A *p*-value of less than 0.05 was considered statistically significant.

## 3. Results

### 3.1. Socio-Demographic Characteristics

All study participants were male and had migrated from southern Mexican states. The majority of the participants came from the state of Chiapas (90%) to work in Sonora (Table 2). The participants’ ages ranged from 19 to 49 years with a median age of 23.5 years and a mean of 26.4 years. More than half of the participants spoke Spanish only, while 45% spoke Spanish in addition to an indigenous language. More than half were married or lived with a common-law partner (55%). With respect to educational attainment, the majority of participants had attended/concluded middle school (45%), several had attended high school (30%), and several had attended elementary school only (25%).

### 3.2. Self-Reported Pesticide Exposure and Work Activities

As presented in Table 3, the majority of participants had more than five years of experience working in agriculture (55%), while 45% of participants had worked in agriculture for less than five years. Most of the participants (80%) reported that they had been employed for less than 6 months at the grape farm where this study took place. While the majority of participants (70%) had not received any training on how to prevent and/or reduce pesticide exposure, 60% of participants stated their employer had suggested wearing some type of protective clothing during fieldwork. Only 25% of participants knew if pesticides were applied in the field and none of the participants knew whether pesticides had been applied in their dormitories on the farm. Approximately 50% of participants stated that they sometimes wore their unwashed work clothes for two consecutive days. The majority of participants (70%) reported that they always washed their work clothing with their leisure clothing.

### 3.3. Urinary Pesticide Metabolite Concentrations

At least one OP and one pyrethroid metabolite were detected in each of the 20 urine samples analyzed. We detected TCPY in 19 (95%) samples, PNP in all 20 samples (100%), 4F3PBA and 3PBA in all 20 (100%) samples, while t-DCCA was detected in 14 (70%) of the samples (Table 4). In addition, the concentrations of the OP and pyrethroid urine metabolites after adjusting for creatinine are shown in Table 4. Among the OP metabolites, the geometric mean for TCPY was higher than PNP (3.56 µg/g and 1.63 µg/g of creatinine, respectively). For the pyrethroid metabolites, 3PBA was higher than 4F3PBA and t-DCCA (1.83 µg/g, 0.84 µg/g, 0.88 µg/g, respectively). As shown in Table 5, the urine metabolite concentrations for the OPs (TCPY and PNP) and pyrethroids (3PBA) detected in our study were higher than in Mexican Americans and significantly higher (*p* < 0.001) than the U.S. general population from NHANES 2009–2010. In addition, urine concentrations for 4F3PBA were detected in all of our samples, but were not in NHANES. The t-DCCA concentrations in our study are only comparable at the NHANES 95^th^ percentile (Table 5; Figures S1–S4 in Supplementary Materials).

### 3.4. Associations between Pesticide Metabolite Concentrations and Socio-Demographic and Occupational Characteristics

As presented in Table 6, for the OPs, a negative correlation was found between the participants’ age and creatinine-adjusted TCPY (Pearson correlation, coefficient value = −0.52, *p* = 0.02). With respect to the pyrethroid metabolites, we found that participants who did not receive personal protective equipment (PPE) training had significantly higher 4F3PBA concentrations than those who reported having received training (*p* = 0.025). Additionally, significantly higher concentrations of 4F3PBA (*p* = 0.019) were found among participants who worked in the grape field for less than three months compared to those who worked in this field for more than three months. Additionally, participants who reported Spanish as well as an indigenous language as their primary languages had higher concentrations of 4F3PBA than those who reported only Spanish as their primary language (*p* = 0.021). Finally, we found higher concentrations among all pesticide metabolites during the summer season, but only 4F3PBA (*p* = 0.005) and 3PBA (*p* = 0.001) were significantly higher during the summer compared to the spring season. Other socio-demographic and occupational pesticide exposure characteristics including education, marital status, experience working in agriculture, knowledge of pesticides applied in the field and dorms, training on pesticide exposure, and washing working clothes with leisure clothing, were not significantly associated with the pesticide metabolite concentrations in our study participants.

## 4. Discussion

In the present study, exposure to OPs and pyrethroids were assessed for the first time in a vulnerable migrant worker population. Median urinary concentrations of the OP and pyrethroid biomarkers were significantly higher in these farmworkers than among Mexican Americans or the U.S. general population from NHANES 2009–2010. These results suggest that these migrant farmworkers’ exposure to chlorpyrifos, parathion, and several pyrethroids is higher than Mexican Americans’ background exposures. Although there is no information for the general population in Mexico for comparison purposes, we hypothesize that the general population of Mexico will have different pesticide biomarker concentrations than the U.S. general population because of differences in pesticide usage, policies and enforcement, dietary intake, and cultural and structural differences [26,27].

The results from this study also suggest higher pesticide exposure in this group of migrant farmworkers compared to other farmworker studies. Only one previous study has evaluated metabolites for chlorpyrifos and parathion, both OPs, in the urine of field workers in Sonora, Mexico. The highest values for chlorpyrifos and parathion metabolites reported by Aldana-Madrid et al. [32] were lower than those in this exploratory study (Table 5). However, Aldana-Madrid et al. [32] studied local field workers from Sonora, which are not representative of our study population, that frequently migrate from the Southern Mexican States and temporarily reside in migrant dormitories located within the farm where they are employed. Interestingly, when comparing our OP results with other farmworker pesticide studies outside of the Sonora region, we found that concentrations of the chlorpyrifos metabolite (TCPY) in our study participants were higher than those reported in studies conducted in Ecuador, Thailand, and a study conducted in Latino farmworkers from North Carolina [38,39,41]. In addition, similar TCPY concentrations were reported in another study from North Carolina in male migrant tobacco workers who did not participate in any pesticide application processes [41]. Parathion metabolite, PNP, concentrations detected in our study were equivalent to the concentrations found in Thailand by Panuwet et al. [39], but lower than the concentrations found by Raymer et al. [41] in North Carolina.

To the best of our knowledge, no other study has evaluated the urine concentrations of pyrethroid metabolites in a migrant Mexican population. In comparison to studies outside of Sonora, urinary concentrations of 3PBA were higher than the results obtained from the North Carolina studies by Raymer et al. [41] and Arcury et al. [38] as well as those reported by Panawet et al. [38] in Thailand and a study conducted by Handal et al. [40] in Ecuador with rose-workers (Table 5). Meanwhile, urinary concentrations for 4F3PBA and t-DCCA in our exploratory study were higher than the results reported by Handal et al. [40] in Ecuador, but they were not reported in the other farmworker studies [38,39,41] (Table 5).

### 4.1. Possible Sources of Exposure to Detected Pesticides

For the general population, dietary intake may account for a significant proportion of pesticide exposure [42,43,44]. To estimate the dietary exposure to pesticides, national databases of pesticide residues and food consumption are commonly utilized; however, many databases lack the capability to select foods based on cultural and socio-demographic backgrounds [45]. In Mexico, assessing the human exposure to pesticides via dietary intake is complicated because there are no official databases on pesticide residues in agricultural products and the database for daily Mexican dietary consumption data is incomplete [46]. Only one study has evaluated the pesticide residues of pyrethroids in legumes and vegetables consumed and produced in Sonora [46]. According to Aldana-Madrid et al. [46], the quantified pyrethroid residues found in the daily Sonoran diet are rather low, suggesting that diet may not represent a significant source of exposure. Thus, the urinary metabolite concentrations of pyrethroids found in our study participants might not have a relatively large contribution from dietary intake. In order to distinguish between non-dietary and dietary sources of pesticides, additional research is needed in this region.

The current study participants resided temporally in dormitories provided by farm owners located inside of the farms, and migrant workers might have been exposed to pesticides in dorms through drift, take-home pathway, or/and insecticide application within dorms to control pests such as bedbugs [47,48]. Even though some OPs such as chlorpyrifos have not been used for residential purposes in the U.S., evidence exists of relatively high levels of OPs and pyrethroids inside U.S. farmworker camps [38,49]. Regardless of the pesticides present in farmworkers’ housing in the U.S., there is limited to no information on the housing of Mexico’s migrant farmworkers. Thus, future studies in Sonora could consider measuring the number of pesticides in migrant workers’ dormitories.

In addition, there is the possibility that some pesticide metabolites found in this migrant population may have not necessarily come from this grape farm in Sonora, but from a previous exposure when working at another farm in their state of origin or from pesticides applied in their residence. Due to the high octanol/water partition coefficient of pyrethroid and OP pesticides, the build-up of these pesticides in fat tissues may have occurred [5,50]. Even though the majority of our study participants (80%) had only been working at this particular farm for less than 6 months, most (55%) had worked in the agricultural industry for more than five years, which may lead to a long-term chronic exposure to pesticides from their diet or/and occupational activities. It is important to consider that the study participants may have been simultaneously exposed to pyrethroids and OPs at some point, resulting in pesticides interactions within the body that may have reduced/increased the detected urine metabolite concentrations [51]. There is evidence to suggest that the co-exposure of some of the pesticides evaluated in this study can produce potentiation or/and additive effects, which can lead to a decreased urinary concentration of 3PBA and an increased tissue concentration of chlorpyrifos and cypermethrin [51,52].

Our findings suggest that several socio-demographic and occupational characteristics such as the participant’s age, PPE training, time working at this farm, language, and season were significantly associated with several of the pesticide urine metabolite concentrations. For pyrethroids, we found associations with wearing personal protective equipment (PPE), time working in this grape field, primary language, and season. Additionally, participants who had been working at the farm for less than three months had significantly higher 4F3PBA concentrations than participants who had worked in this field for more than three months, which may be related to the workers’ experience gained in this farm, or pesticide application times. Participants who did not receive training on PPE and participants who reported speaking an indigenous language in addition to Spanish had significantly higher urinary concentrations of 4F3PBA. This suggests that many farmworkers may not know how to protect themselves, which coincides with the fact that PPE is not required for workers who do not directly handle pesticides.

Furthermore, the language and cultural barriers that exist between the farmworkers and supervisors/managers/owners combined with the low education attained by workers can aggravate the pesticide risk exposure in migratory farm worker populations [48]. Although the length of time spent working in the agricultural industry was not significantly associated with pesticide metabolite concentrations, the participants’ age was negatively correlated with TCPY urinary concentrations. The negative correlation between age and pesticide exposure has been reported in a previous farmworker study [53]. This association might have happened because pesticides may not be as effectively removed by the body as a person ages since functional changes occur in the liver and kidneys with the increase of age during adulthood [54,55]. While some pesticides are stored in fat tissue before they are eliminated from the body by the kidney and liver, the mass of these organ decreases progressively as a person ages; thus, pesticides cannot be fully filtered and removed in urine and may stay longer in fat tissues [56]. Additionally, the migrant farmworkers who participated in this study are part of a socially vulnerable population who do not necessarily have access to important services such as education. The questionnaire portion of our study revealed that all of the participants migrated from the southern states of Mexico with a relatively low educational attainment (30% reported attending high school). It is important to understand the socio-demographic and occupational exposure characteristics of this studied population because some of these characteristics can be associated with pesticide exposure [26].

Finally, although the working activities were relatively similar during the summer and spring season, we found that participants who worked during the summer season had higher urinary concentrations of 4F3PBA and 3PBA than participants who worked during the spring season. Several factors such as heat and pesticide application times may have influenced the seasonal variation of the urinary concentrations, but these were not evaluated in this study. It is important to note that the northwestern state of Sonora is considered approximately 95% arid or semi-arid land characterized by lack of precipitation and high temperatures. Thus, migrant farmworkers in this region frequently experience a combination of extreme heat conditions and pesticide exposure. Some experimental studies have shown that agrochemical exposure and heat stress, combined with heavy work/exercise can have synergistic effects exacerbating their negative health effects [57,58].

### 4.2. Limitations

Key limitations of our study include its cross-sectional design, which limits our ability to establish temporal order. Having a convenience sample can create a sample bias since participants who have agreed to be in the study may have different socio-demographic risk potentials and occupational health knowledge than those who were not part of the study. Additionally, these results are difficult to interpret due to the sampling variability associated with the small sample size. Due to limitations in funding, we did not have a control group for comparison with field workers. It is not possible to determine how representative those sampled are of the larger population of migrant workers at this large farm (*n* = 2200). A larger sample size would provide more power to evaluate the associations between the exposure characteristics and the pesticide urine metabolite concentrations. Another potential disadvantage of our study is that we only collected one sample per participant and our samples were collected without knowing the application times and quantity for all of the target pesticides. In addition, although we recognized that food can be an important source of pesticide exposure, our study lacked a detail information on the food consumption for this population.

## 5. Conclusions

This convenience group of migrant grape workers in Sonora, Mexico had higher urine concentrations of select OP and pyrethroid insecticide biomarkers than Mexican Americans, the U.S. general population, and other farmworkers. This was the first study that evaluated OP and pyrethroid exposure using urine biomarkers in this vulnerable and migrant population. A larger cohort study, incorporating health outcomes, and collecting multiple pesticide bio-monitoring measurements could improve the understanding of pesticide exposure in this region. Specifically, further research at a population level would help to better understand the general population’s exposure to pesticides in this region, characterize migrant farmworkers’ exposures to pesticides, and explore the possible sources of pesticide exposure related to agricultural activities and the possible interactions between heat and pesticide exposure. We also report that several occupational and socio-demographic characteristics such as age, PPE training, time working at the farm, language, and season are potential exposure determinants. Further research with a larger sample size can help to better understand and define key elements of pesticide exposure in this population, as well as possible interventions and occupational safety programs that may help reduce exposure to pesticides in this region.

## Figures and Tables

**Table 1:** The pesticide biomarkers measured in urine.

Biomarker	Abbreviation	Parent Chemical(s)	Pesticide Class	LOD
4-fluoro-3-phenoxybenzoic acid	4F3PBA	Cyfluthrin	Pyrethroid	0.1 µg/L
trans-3-(2,2-Dichlorovinyl)-2,2-dimethylcyclopropane carboxylic acid	t-DCCA	Permethrin; Cypermethrin; Cyfluthrin	Pyrethroid	0.6 µg/L
3-phenoxybenzoic acid	3PBA	Cyhalothrin, Cypermethrin, Deltamethrin, Fenpropathrin, Permethrin, Tralomethrin	Pyrethroid	0.1 µg/L
para-Nitrophenol	PNP	Parathion; Methyl parathion	OP	0.1 µg/L
3,5,6-Trichloro-2-pyridinol	TCPY	Chlorpyrifos, Chlorpyrifos-methyl	OP	0.1 µg/L

Abbreviation: LOD, limit of detection; OP, organophosphate.

**Table 2:** The socio-demographic characteristics (*N* = 20).

	Min	Max	Median	Mean	SD
Age (years)	19	49	23.5	26.4	7.04
		Frequency (N)		Percent (%)	
**Home language**					
Spanish only		11		55	
Spanish & indigenous language		9		45	
**State of origin**					
Chiapas		18		90	
Veracruz		1		5	
Tabasco		1		5	
**Gender**					
Male		20		100	
**Marital Status**					
Single		9		45	
Married		11		55	
**Education**					
Some Elementary school		5		25	
Some Middle school		9		45	
Some High school		6		30	

Abbreviations: N, number; SD, standard deviation; Min, minimum; Max, maximum.

**Table 3:** The pesticide exposure of participants (*N* = 20) based on self-reported data.

Pesticide Exposure (Questionnaire Responses)	Frequency (N)	Percent (%)
**Agricultural experience**		
Less than 1 year	5	25
Between 1 year to 5 years	4	20
More than 5 years	11	55
**Time working in this particular grape field**		
Less than or equal to 3 months	7	35
More than 3 months	13	65
**Knows which pesticides are applied in the fields**		
No	15	75
Yes	5	25
**Knows if pesticides have been applied in dorms**		
No	20	100
Yes	0	0
**Received training on how to reduce/prevent pesticide exposure in this**		
**field**		
No	14	70
Yes	6	30
**Received training on types of protective clothing**		
No	8	40
Yes	12	60
**Wear the same work clothing for more than two consecutive days without**		
**washing**		
Always	6	30
Sometimes	10	50
Never	4	20
**Wash work clothing with leisure clothing**		
Always	14	70
Sometimes	3	15
Never	3	15

Abbreviations: N, number; %, percent.

**Table 4:** The creatinine-adjusted urine metabolite concentrations (*N* = 20).

	Frequency (*N*)	Detection (%)	Range (µg/g of Creatinine)	GM (µg/g of Creatinine)	GSD (µg/g of Creatinine)
Organophosphates metabolites					
TCPY	19	95	0.07–13.24	3.56	2.85
PNP	20	100	1.06–2.87	1.63	1.37
Pyrethroid metabolites					
4F3PBA	20	100	0.26–7.05	0.94	2.76
3PBA	20	100	0.90–4.96	1.83	1.70
t-DCCA	14	70	0.42–4.00	0.88	2.30

Abbreviations: *N*, number; %, percent; SD, standard deviation; GM, geometric mean. TCPY: 3,5,6-trichloro-2-pyridinol; PNP: para-nitrophenol; 4F3PBA: 4-fluoro-3-phenoxybenzoic acid; 3PBA: 3-phenoxybenzoic acid; t-DCCA: trans-3-(2,2-dichlorovinyl)-2,2-dimethylcyclopropane carboxylic acid. Values below LOD were substituted by LOD divided by square root of 2. The limits of detection (LODs) were: 0.1 µg/L for all analytes, except t-DCCA (0.6 µg/L).

**Table 5:** The comparison of study results for urine metabolites with results from other studies.

Metabolite	Study	Frequency (N)	Detection (%)	LOD (µg/L)	Geometric Mean (µg/g)	Percentile		
						50^th^	75^th^	95^th^
TCPY	*Current Study*	19	95	0.1	3.56	3.63	5.67	10.82
	*Current Study* ^ⱡ^	19	95	0.1	4.17	4.43	7.19	13.6
	NHANES Total (*n* = 2747) *	1923	70	0.1	0.81	0.98	1.66	3.53
	NHANES Mex American (*n* = 602)			0.1	0.76	0.95	1.9	4.64
	Arcury et al. [38]	112	100	0.2	3.3	3	6.94	15.02
	Panuwet et al. [39]	107	77	0.2	1.3	1.3	3.5	20.6
	Handal et al. [40] ^ⱡ^	14	86	0.1	0.94	1.04		11.0
	Raymer et al. [41] ^ⱡ^	160	44		4.5			
	Aldana-Madrid et al. [32] ^ⱡ^	15	28	0.1				3.40 ^+^
	*Current Study*	20	100	0.1	1.63	1.62	2.07	2.69
	*Current Study* ^ⱡ^	20	100	0.1	1.91	1.84	3.05	4.53
	NHANES Total (*n* = 2744) *	2113	77	0.1	0.47	0.49	0.92	2.62
PNP	NHANES Mex American (*n* = 602)			0.1	0.51	0.54	1.02	2.35
	Panuwet et al. [39]	135	99	0.1	2.1	2.2	2.9	4.7
	Raymer et al. [41] ^ⱡ^	220	61		2.94			
	Aldana-Madrid et al. [32] ^ⱡ^	4	7	0.1				2.00 ^+^
3PBA	*Current Study*	20	100	0.1	1.83	1.69	2.39	4.65
	*Current Study* ^ⱡ^	20	100	0.1	2.14	2.14	2.86	5.55
	NHANES Total (*n* = 2747) *	2205	81	0.1	0.44	0.38	1.01	5.44
	NHANES Mex American (*n* = 602)			0.1	0.39	0.36	0.7	3.22
	Arcury et al. [38]	107	96	0.4	1.03	1.04	1.7	3.16
	Panuwet et al. [39]	118	87	0.1	0.86	0.98	2.5	7.4
	Handal et al. [40] ^ⱡ^	6	35	0.1	0.12	<LOD		3.93
	Raymer et al. [41] ^ⱡ^	154	43		2.29			
t-DCCA	*Current Study*	14	70	0.6	0.88	0.9	1.41	3.65
	NHANES Total (*n* = 2747)	28	0.1	0.6	NC	<LOD	<LOD	4.37
	NHANES Mex American (*n* = 602)			0.6	NC	<LOD	<LOD	2.57
	Handal et al. [40] ^ⱡ^	1	6	0.1	0.10	<LOD	<LOD	16.59
	Panuwet et al. [39]	51	38	0.2	NC	<LOD	1.9	11.1
4F3PBA	*Current Study*	20	100	0.1	0.94	0.51	2.52	4.51
	NHANES Total (*n* = 2747)	ND	0	0.1	NC	<LOD	<LOD	<LOD
	NHANES Mex American (*n* = 602)	ND	0	0.1	NC	<LOD	<LOD	<LOD
	Raymer et al. [41] ^ⱡ^	19	5		NC			

^ⱡ^ Not creatinine-adjusted (μg/L for non-creatinine-adjusted results). * Metabolite concentrations from our study were significantly higher than the general U.S population, NHANES 2009–2010, (Mann–Whitney *U* test at *p* < 0.001). Note: NHANES dataset includes males and females of all age groups. ^+^ Maximum urine metabolite values found in the study. Abbreviations: N, number; TCPY: 3,5,6-trichloro-2-pyridinol; PNP: para-nitrophenol; 4F3PBA: 4-fluoro-3-phenoxybenzoic acid; 3PBA: 3-phenoxybenzoic acid; t-DCCA: trans-3-(2,2-dichlorovinyl)-2,2-dimethylcyclopropane carboxylic acid. NC: values were not provided by the original study because geometric means could not be calculated due to the small number of detections. ND: not detected.

**Table 6:** The urine metabolite concentrations (µg/g of creatinine) and pesticide exposure characteristics.

Characteristics (*N*)	TCPY		PNP		3PBA		t-DCCA		4F3PBA	
	GM	(*p*-Value)	GM	(*p*-Value)	GM	(*p*-Value)	GM	(*p*-Value)	GM	(*p*-Value)
**Time in this grape field**										
<3 months (7)	3.25	(0.053)	1.71	(0.607)	2.21	(0.322)	1.06	(0.552)	2.13	(0.019) *
>3 months (13)	3.74		1.59		1.65		0.80		0.61	
**Language**										
Spanish only (11)	3.10	(0.676)	1.57	(0.621)	1.51	(0.063)	0.84	(0.970)	0.55	(0.021) *
Spanish & Ind. (9)	4.22		1.71		2.30		0.91		1.80 ^ɫ^	
**Training on PPE**										
Yes (12)	3.73	(0.054)	1.61	(0.757)	1.70	(0.487)	0.79	(0.537)	0.62	(0.025) ^ϒ^
No (8)	3.32		1.66		2.05		1.04		1.78	
**Season**										
Spring (10)	2.82	(0.880)	1.67	(0.597)	1.23	(0.001) ^ⱡ^	0.74	(0.364)	0.47	(0.005) ^ⱡ^
Summer (10)	4.50		1.59		2.71		1.06		1.90	
	**Spearman’s Correlation Coefficient (*p*-value)**									
**Participants age (20**)	−0.52 (0.02) **		0.06 (0.79)		−0.29 (0.21)		−0.16 (0.49)		−0.17 (0.46)	

Abbreviations: N, number; GM, geometric mean; TCPY: 3,5,6-trichloro-2-pyridinol; PNP: para-nitrophenol; 4F3PBA: 4-fluoro-3-phenoxybenzoic acid; 3PBA: 3-phenoxybenzoic acid; t-DCCA: trans-3-(2,2-dichlorovinyl)-2,2-dimethylcyclopropane carboxylic acid; PPE: personal protective equipment. * The significant difference was observed (Mann–Whitney *U* test at *p*-value < 0.05) for 4F-3PBA between the participants whose home language is only Spanish versus participants whose home language is Spanish and an indigenous language; in addition to participants who have worked in this field longer or less than 3 months. ^ϒ^ A significant difference was observed for the 4F3PBA (Mann–Whitney *U* test *p*-value < 0.05) between the participants who have received training from employer to wear some type of PPE, and participants who did not receive any suggestion from the employer. ^ⱡ^ A significant difference was observed for the 4F-3PBA (*p*-value = 0.05) and 3PBA (*p* = 0.001) between the participants who have worked in this grape field during the spring versus the summer season. ** Significant moderate negative correlation between age and TCPY (Spearman’s correlation = −0.52, *p* < 0.05)

## Supplementary Materials

In Figures S1 and S2, our findings suggest that TCPY, PNP, and 3PBA urine concentrations seem to be higher than among Mexican Americans (*n* = 602) obtained from NHANES 2009–2010. 4F3PBA and t-DCCA were detected in our study, but not in the Mexican Americans. Additionally, the urine concentration distributions in our pilot study were significantly higher than the U.S. general population (NHANES 2009–2010) for TCPY (KS-test = 0.808, *p* 0.001), PNP (KS-test = 0.789, *p* < 0.001), 3PBA (KS-test = 0.738, *p* < 0.001), and t-DCCA (KS-test = 0.378, *p* < 0.001). The urine metabolite of cyfluthrin (F3PBA) was only detected in our study, but not in the general U.S. population (please see Figures S3 and S4 in supplementary material).
