# Supplementary material for: Migrant Farmworkers’ Exposure to Pesticides in Sonora, Mexico
Source: Int J Environ Res Public Health. 2018 Nov 26;15(12):2651. doi: 10.3390/ijerph15122651 (PMC6313604; doi:10.3390/ijerph15122651)
Supplement: Supplementary file 1 [file ijerph-15-02651-s001.pdf]

International Journal of
Environmental Research and Public Health

Supplementary Materials: Migrant Farmworkers' Exposure to Pesticides in Sonora, Mexico

Nicolás López-Gálvez *, Rietta Wagoner , Paloma Beamer , Jill de Zapien and Cecilia Rosales

Mel and Enid Zuckerman College of Public Health, University of Arizona, 1295 N. Martin Ave. PO 245210, Tucson, AZ 85724, USA

; rwagoner@email.arizona.edu (R.W.); pbeamer@email.arizona.edu (P.B.); dezapien@email.arizona.edu (J.d.Z.); crosales@email.arizona.edu (C.R.)

* Correspondence: lopezgalvez@email.arizona.edu; Tel.: +01-619-718-0794

Received: 3 November 2018; Accepted: 20 November 2018; Published: 26 November 2018

In Figures S1 and S2, our findings suggest that TCPY, PNP, and 3PBA urine concentrations seem to be higher than among Mexican Americans (n = 602) obtained from NHANES 2009–2010. 4F3PBA and t-DCCA were detected in our study, but not in the Mexican Americans.

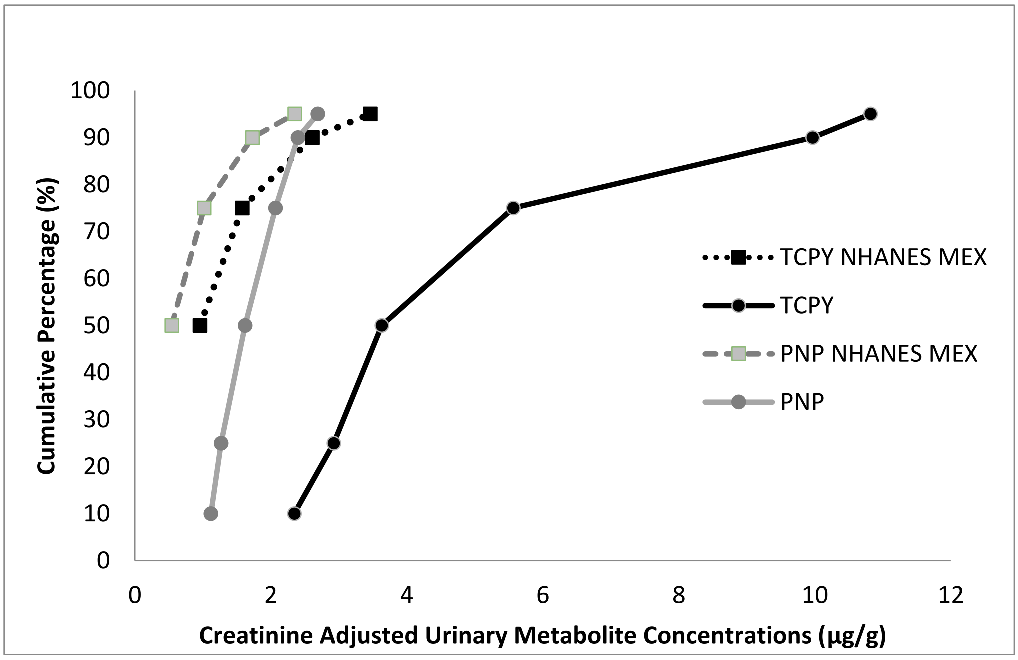

Figure S1. Organophosphate urine metabolite concentrations in migrant grape workers of Sonora, Mexico, compared to the Mexican American population (N = 602) from NHANES 2009–2010. Abbreviations: TCPY NHANES MEX: urinary 3,5,6-trichloro-2-pyridinol concentrations from the Mexican-American population of the NHANES 2009–2010; TCPY: urinary 3,5,6-trichloro-2-pyridinol concentrations from participants in this study, PNP NHANES MEX: urinary para-nitrophenol concentrations from the Mexican-American population of the NHANES 2009–2010, PNP: urinary para-nitrophenol concentrations from participants in this study.

International Journal of
Environmental Research and Public Health
MDPI

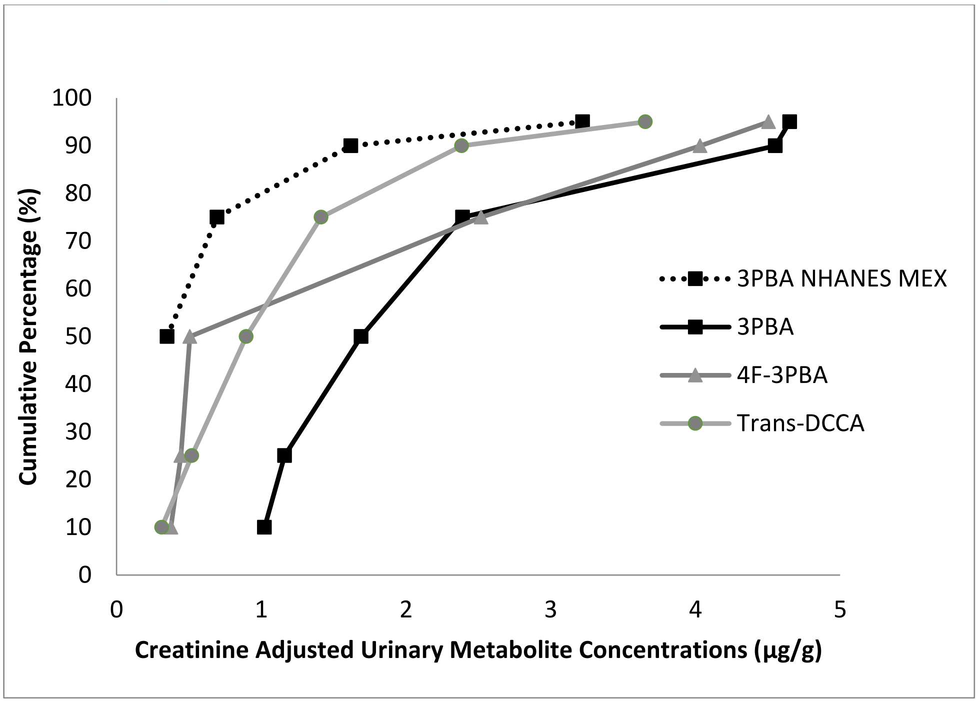

Figure S2. Pyrethroid urine metabolite concentrations in migrant grape workers of Sonora, Mexico, compared to the Mexican American population (N = 602) from NHANES 2009–2010. Abbreviations: 4F3PBA: 4-fluoro-3-phenoxybenzoic acid; 3PBA: 3-phenoxybenzoic acid; Trans-DCCA: trans-3-(2,2-dichlorovinyl)-2,2-dimethylcyclopropane carboxylic acid. Note: the metabolite concentrations for 4F3PBA and t-DCCA found in this study were not detected in NHANES (< LOD).

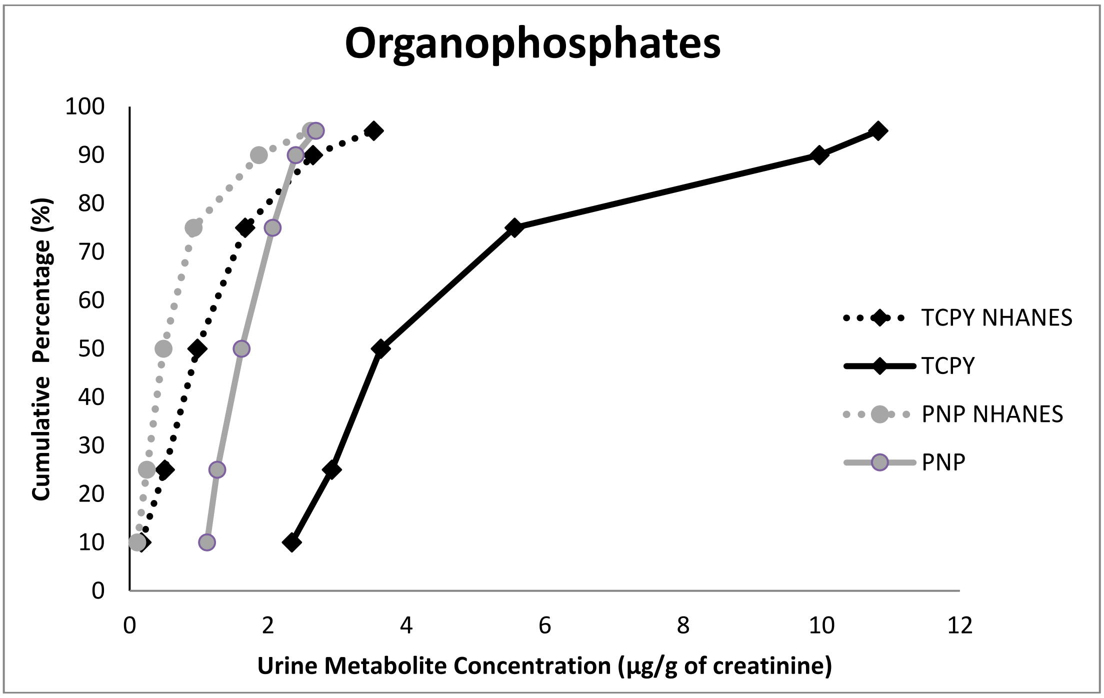

Figure S3. Organophosphate urine metabolite concentrations in migrant grape workers of Sonora, Mexico, compared to the total population (N = 2,747) from NHANES 2009-2010. Abbreviations: TCPY: 3,5,6-trichloro-2-pyridinol; PNP: para-nitrophenol.

International Journal of
*Environmental Research
and Public Health*

MDPI

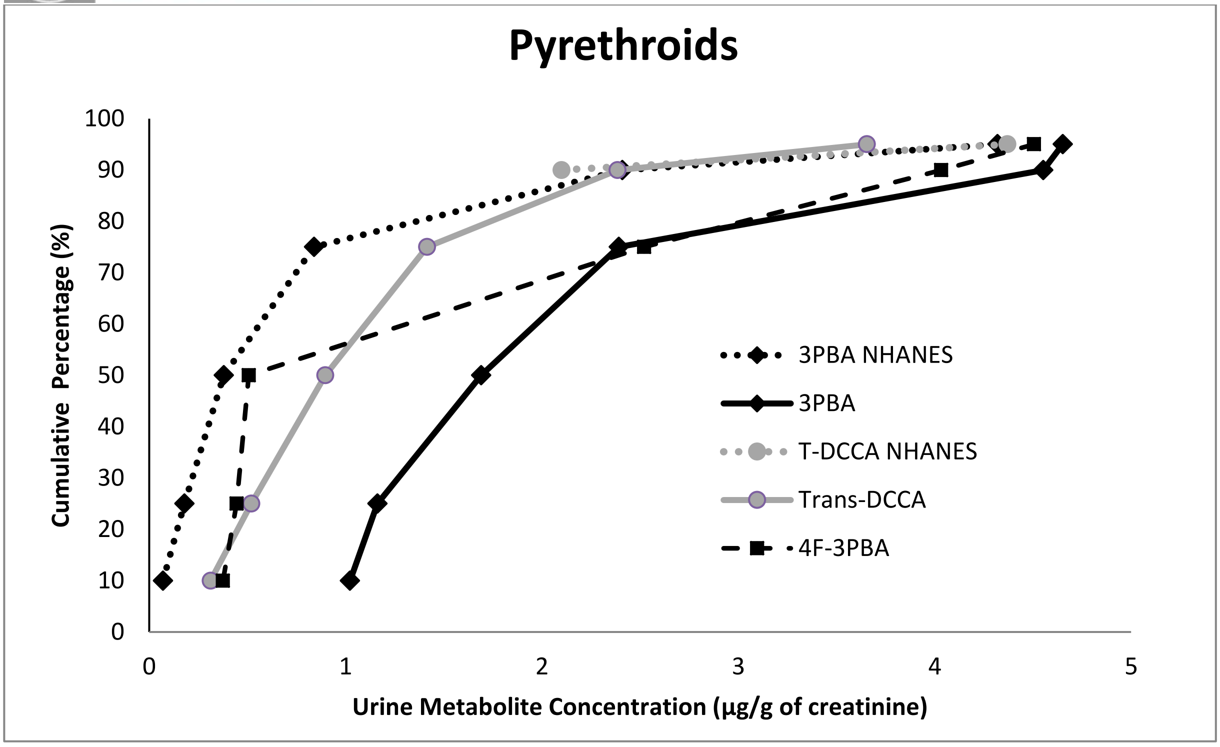

Figure S4. Pyrethroid urine metabolite concentrations in migrant grape workers of Sonora, Mexico, compared to the with NHANES 2009 -2010 total population (*N* = 2,747). Abbreviations: 4F3PBA: 4-fluoro-3-phenoxybenzoic acid; 3PBA: 3-phenoxybenzoic acid; t-DCCA: trans-3-(2,2-dichlorovinyl)-2,2-dimethylcyclopropane carboxylic acid. Note: the metabolite concentrations for 4F3PBA found in this study were not detected in NHANES (< LOD).

As presented in Figure S3 , the organophosphates urine metabolite concentrations in our study were higher than the total U.S. population. The urine concentration distributions in our pilot study were significantly higher than NHANES 2009-2010 for TCPY (KS-test = 0.808, $p < 0.001$), and PNP (KS-test = 0.789, $p < 0.001$). As presented in Figure S4, the pyrethroids distributions of the urine concentrations found in our study were significantly higher than NHANES for 3PBA (KS-test = 0.738, $p < 0.001$), t-DCCA (KS-test = 0.378, $p < 0.001$). In addition, the urine metabolite of cyfluthrin (F3PBA) was only detected in our study, but not in the NHANES 2009-2010 U.S. population.
